# Impairments in Neurogenesis Are Not Tightly Linked to Depressive Behavior in a Transgenic Mouse Model of Alzheimer's Disease

**DOI:** 10.1371/journal.pone.0079651

**Published:** 2013-11-14

**Authors:** Daniel M. Iascone, Sneha Padidam, Mark S. Pyfer, Xiaohong Zhang, Lijuan Zhao, Jeannie Chin

**Affiliations:** Department of Neuroscience and Farber Institute for Neurosciences, Thomas Jefferson University, Philadelphia, Pennsylvania, United States of America; Universidad de Sevilla, Spain

## Abstract

Alzheimer's disease (AD), the most common cause of dementia, is also associated with depression. Although the precise mechanisms that lead to depression in AD are unknown, the impairments in adult hippocampal neurogenesis observed in AD may play a role. Adult-born neurons play a critical role in regulating both cognition and mood, and reduced hippocampal neurogenesis is associated with depression in other neurological disorders. To assess the relationship between Alzheimer's disease, neurogenesis, and depression, we studied human amyloid precursor protein (hAPP) transgenic mice, a well-characterized model of AD. We report that reductions in hippocampal neurogenesis are evident early in disease progression in hAPP mice, but a mild depressive phenotype manifests only in later stages of disease. We found that hAPP mice exhibited a reduction in BrdU-positive cells in the subgranular zone of the dentate gyrus in the hippocampus, as well as a reduction in doublecortin-expressing cells, relative to nontransgenic controls at 5–7 months of age. These alterations in neurogenesis appeared to worsen with age, as the magnitude of reduction in doublecortin-expressing cells was greater in hAPP mice at 13–15 months of age. Only 13–15 month old hAPP mice exhibited depressive behavior in the tail suspension test. However, mice at both age groups exhibited deficits in spatial memory, which was observed in the Morris water maze test for hippocampus-dependent memory. These findings indicate that neurogenesis impairments are accompanied by cognitive deficits, but are not tightly linked to depressive behavior in hAPP mice.

## Introduction

Alzheimer's disease (AD) is the most common form of dementia and is characterized by prominent impairments in memory [1]. The amyloid precursor protein and the amyloid-β (Aβ) peptides cleaved from it play central roles in AD [2], [3]. Aβ is prone to aggregation, and deposits into extracellular plaques that are pathological hallmarks of AD. However, smaller Aβ oligomers disrupt neuronal function early in disease progression, before plaques begin to form in mouse models of AD [3], [4]. The precise mechanisms through which Aβ impairs neuronal function are unclear.

Neurogenesis is also altered early in disease progression, prior to plaque deposition in AD mouse models, suggesting that it could play a role in early cognitive and/or psychiatric symptoms of AD [5]–[8]. In adult organisms, neurogenesis occurs in only two specific brain regions, one of which is the subgranular zone of the hippocampus [9]. The hippocampus is critical for learning and memory as well as mood regulation, and adult neurogenesis is necessary for normal function [9]–[11]. Notably, the hippocampus is also particularly vulnerable in AD [12], [13]. A number of reports have demonstrated that adult neurogenesis is altered in AD patients as well as mouse models; both increases and decreases in neurogenesis have been reported which may reflect different stages of disease [5]–[7], [14].

Several studies have examined the implications of impaired neurogenesis for cognitive deficits in AD, but few have examined whether neurogenesis impairments are related to the increased incidence of depression observed in AD [7], [15]. Experimentally reduced neurogenesis alone is not sufficient to induce depressive behavior in rodents, although rodent models of depression are associated with deficits in adult neurogenesis [16], [17]. Moreover, the therapeutic effects of certain classes of antidepressants rely upon mechanisms that increase neurogenesis [16]–[18]. Thus, there is a complex relationship between alterations in neurogenesis and depression [19]. Because neurogenesis regulates critical functions in the adult brain, and is affected early in AD [7], [10], [11], [14], it is important to understand the consequences of impaired neurogenesis to AD pathophysiology.

We investigated the relationship between impairments in neurogenesis and development of depressive behavior in transgenic mice that overexpress human amyloid precursor protein carrying AD-associated mutations (hAPP mice), a well-characterized model of AD. We assessed depressive behavior using the tail suspension test, and analyzed hippocampal neurogenesis using BrdU to label dividing cells as well as immunophenotyping to identify and count doublecortin-expressing cells. We examined hAPP mice at two ages, and found that aged hAPP transgenic mice exhibit both impaired neurogenesis and depressive behavior, but that deficits in neurogenesis develop well before depressive behavior is observed. Thus, impairments in hippocampal neurogenesis are not tightly linked to depressive behavior in hAPP transgenic mice.

## Materials and Methods

### Ethics Statement

All animal work was performed with the approval of the Thomas Jefferson University Institutional Animal Care and Use Committee, under protocol #884A.

### Transgenic Mice

hAPP transgenic line J20 heterozygously expresses hAPP carrying the Swedish (K670N, M671L) and Indiana (V717F) familial AD (FAD) mutations (hAPP770 numbering). Transgene expression in this line is directed by the platelet-derived growth factor β chain promoter (Mucke et al., 2000). This line has been crossed for >10 generations onto a C57BL/6 background using nontransgenic (NTG) C57BL/6 mice from The Jackson Laboratory (Bar Harbor, ME). hAPP mice are bred, maintained, and analyzed as heterozygous transgenic mice, and age-matched NTG littermates from the same line are used as controls. Before behavioral testing, mice were singly housed and allowed to acclimate for at least 4 days prior to testing. Age-matched hAPP and NTG mice were evaluated at 5–7 months of age (n = 12/genotype for doublecortin expression studies, n = 6–7/genotype for BrdU experiments, n = 12/genotype for water maze studies, n = 24/genotype for depression studies) and 13–15 months of age (n = 11–12/genotype for doublecortin expression, BrdU, and depression studies, n = 7–11 for water maze studies). Cohorts of mixed sex (containing equivalent numbers of male and female mice) were used for depression and neurogenesis experiments, no sex differences were found. Two separate cohorts of male mice were used for water maze experiments. All behavioral experiments took place during the light phase. At least three days after behavioral studies were complete, mice were anesthetized and flush-perfused transcardially with PBS. Brains were fixed in 4% phosphate-buffered paraformaldehyde.

### BrdU labeling

BrdU (150 mg/kg, Sigma, St. Louis, MO) was dissolved in saline and administered intraperitoneally twice, spaced 2 hours apart. Mice were sacrificed two hours after the last BrdU injection. Brain sections were stained with rat-anti-BrdU antibody (Accurate, Westbury, NY) followed by donkey-anti-rat secondary antibody conjugated to AlexaFluor-594 (Invitrogen, Grand Island, NY). Prolong Gold antifade mounting medium with DAPI (Invitrogen) was used to allow visualization of nuclei. Labeling was visualized with epifluorescence microscopy. BrdU-positive cells in the subgranular zone of the hippocampus were counted in every 10th coronal section throughout the dorsal-ventral extent of the hippocampus, and summed.

### Immunohistochemistry

Sliding microtome sections (30 µm) were avidin-biotin/immunoperoxidase stained using an anti-doublecortin antibody (Cell Signaling, Danvers, MA) followed by biotinylated goat anti-rabbit antibody (Vector Laboratories, Burlingame, CA). Diaminobenzidine was used as the chromagen. Doublecortin-immunoreactive neurons in the subgranular zone of the hippocampus were counted in every 10th coronal section throughout the dorsal-ventral extent of the hippocampus, and summed. Neuroblasts were distinguished from immature neurons on the basis of morphology [20], [21].

### Tail Suspension Test

Mice were suspended 53 cm above a cushioned pad, using tape to attach their tails to a horizontal pole above the pad (protocol adapted from [22]. A tail restriction device was used to prevent the animals from climbing their tails. Each mouse was tested for a 6 minute trial at baseline and 24 hours later. Latency to the first bout of immobility (defined as ≥5 second-long segment of time spent immobile) and the total time spent immobile during the trial were recorded by two independent experimenters blinded to genotype. Immobility was defined as hanging passively without any movement of the head or paws.

### Morris Water Maze

Male hAPP and NTG mice were housed in the testing room at least 4 days prior to testing in the water maze based on our previously published protocol [23], [24]. Mice were tested in a white circular pool (1.2 m diameter) filled with 21–22°C water made opaque with nontoxic white paint. The same platform (10 cm diameter) was used for all phases of the water maze, and was submerged 1.5 cm below the surface of the water. Before testing began, mice were pre-trained for one day by placing them on the platform in the middle of the pool. Mice typically jumped off the platform the first 1–2 times. Once the mouse stayed on the platform after being placed there, the next two trials consisted of placing the mouse in the water at the edge of the pool and letting the mouse swim to the platform. For the cued platform trials, a black and white striped rod (2 cm diameter, 25 cm height) was placed vertically in the center of the platform. The distance traveled to reach the platform was measured in two sessions/day for two days. Each session consisted of two trials in which mice were dropped from different locations, and the platform was located in different quadrants, to assess visual acuity and motor performance. 60 s trials were separated by a 15 min inter-trial interval.

Hidden platform training involved two sessions/day for five days. Each session consisted of three trials. 60 s trials were separated by a 15 min inter-trial interval. During acquisition trials, the platform location remained constant and mice entered the pool from randomized locations. 24 hours after the last training trial, mice were given a 60 s probe trial test in which the platform had been removed from the pool. Performance in the cued, hidden, and probe trial portions of the maze was video-tracked and analyzed using WaterMazeScan of the TopScan software suite (CleverSys Inc, Reston, VA). No mice exhibited notable floating or thigmotaxic behavior that affected performance. The experimenter was blinded to genotypes during testing.

### Statistical Analysis

Immunohistochemical data were analyzed using two-tailed, unpaired Student's t-tests. A p-value of less than 0.05 was considered statistically significant. Behavioral data were analyzed with two-way repeated measures ANOVA with Bonferroni post hoc tests to compare the performance of hAPP mice relative to NTG controls in the Morris water maze, as well in minute-by-minute assessment for the tail suspension test. Probe trial performance was assessed using one-way ANOVA followed by Tukey post hoc tests for multiple comparisons. Two-way repeated measures ANOVA was used to compare performances of hAPP and NTG mice in the 24-hour trial of the tail suspension test relative to performance in the baseline trial. One-way ANOVA with Newman-Kuels post hoc tests were used for multiple comparisons tests on the same data.

## Results

### Hippocampal neurogenesis is impaired in hAPP mice at 5–7 months of age

**Figure 1:**
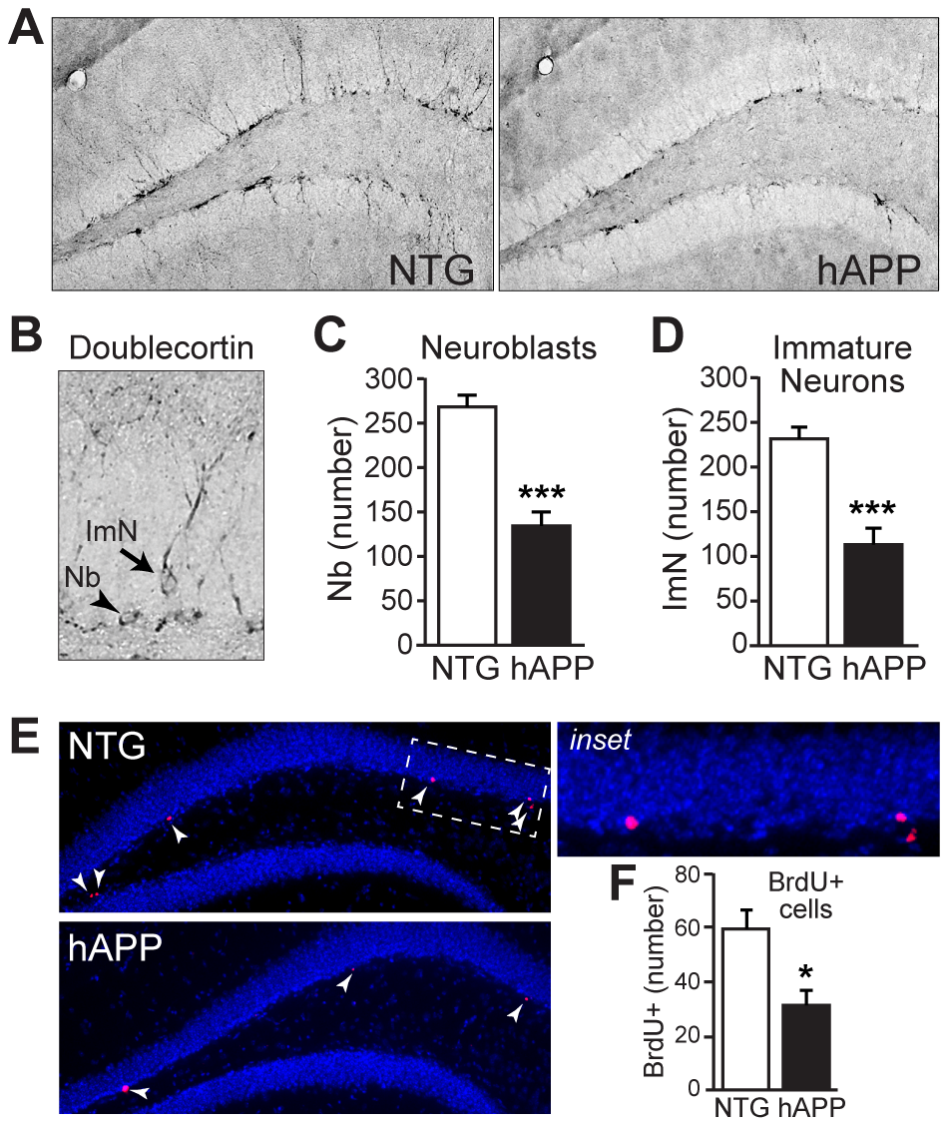
5–7 month old hAPP mice exhibit impairments in neurogenesis. A, Micrographs of doublecortin immunostaining in coronal brain sections from NTG and hAPP mice. B, High magnification micrograph illustrating doublecortin-positive neuroblasts (Nb) and immature neurons (ImN). C–D, Quantification of doublecortin expression demonstrates significant decreases in neuroblasts (C) and immature neurons (D) in hAPP mice relative to NTG controls. E–F, BrdU-labeling of dividing cells in the subgranular zone confirms a decrease in cell division in hAPP mice compared to NTG mice. Arrowheads, BrdU-labeled cells. n = 12/genotype. *p<0.05, ***p<0.001.

We examined doublecortin expression in the dentate gyrus of the hippocampus in coronal sections from hAPP mice at 5–7 months of age, when behavioral/cognitive deficits are robust, but before the development of extracellular plaques [23], [25], [26]. Doublecortin-expressing neuroblasts and immature neurons were distinguished on the basis of morphology [20], [21]. Neuroblasts are only found in the subgranular zone, and appear as cell bodies without any neuronal processes. Immature neurons can be found in either the subgranular zone or the granule cell layer, and have neuronal processes (Fig. 1A–B). We observed significant decreases in numbers of neuroblasts in hAPP mice relative to NTG controls (Fig. 1C, t[22] = 6.701, p<0.001). We also found significant decreases in numbers of immature neurons in hAPP mice relative to NTG controls (Fig. 1D, t[22] = 5.202, p<0.001).

Because the maturation of adult-born neurons is impaired in hAPP mice [27], reductions in doublecortin-positive immature neurons may reflect decreases in cell proliferation and/or the loss of neuroblasts and immature neurons after cell proliferation has occurred. To determine whether cell proliferation is reduced in hAPP mice, we examined the incorporation of BrdU into cells in the subgranular zone of the dentate gyrus of 5–7 month old hAPP and NTG mice. We found that the numbers of BrdU-labeled cells was reduced in hAPP mice relative to NTG controls (Fig. 1E–F; t[11] = 3.076, p = 0.01), demonstrating that neurogenesis is decreased in 5–7 month old hAPP mice.

### hAPP mice do not exhibit depressive behavior at 5–7 months of age

**Figure 2:**
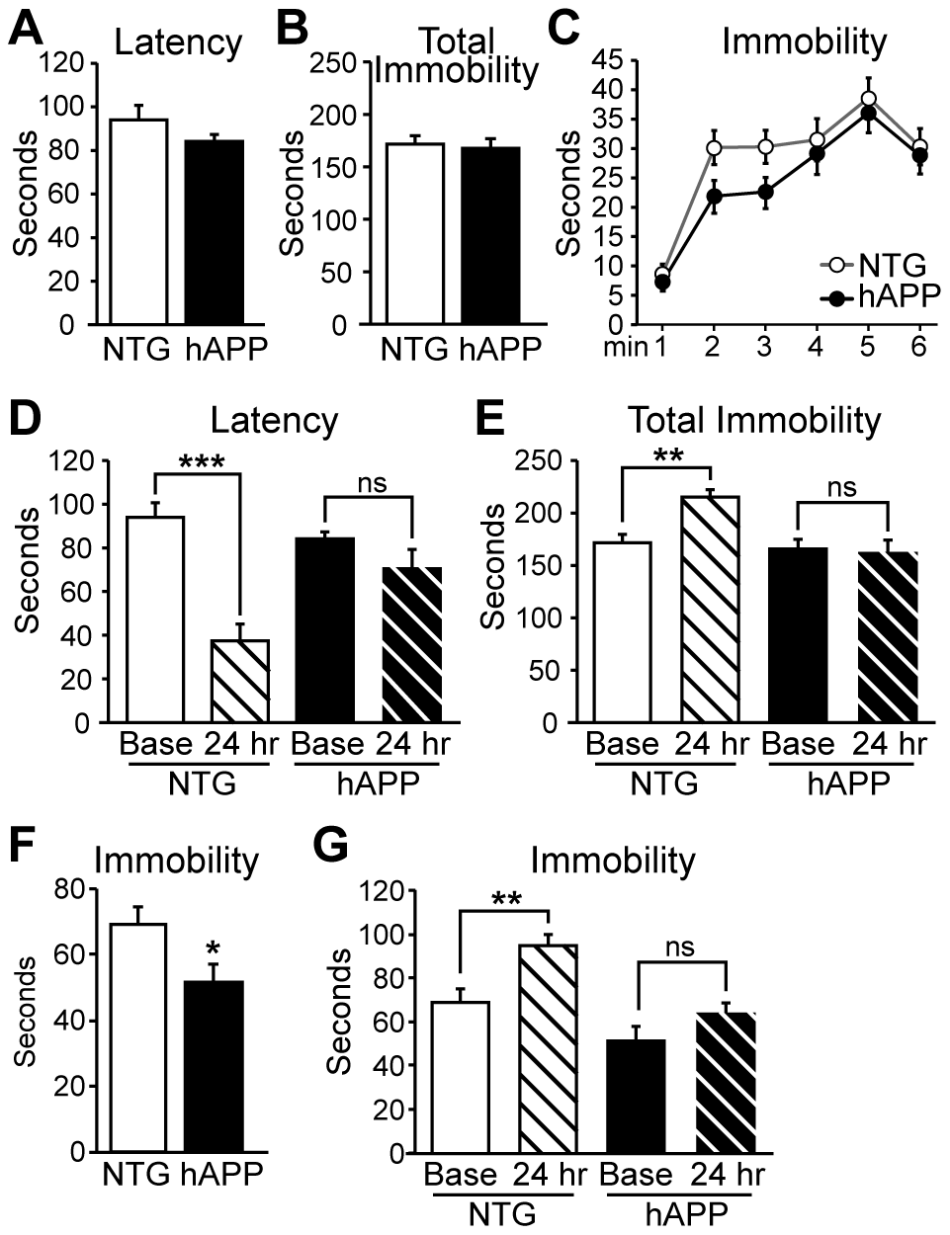
5–7 month old hAPP mice do not display depressive behavior. A, Latency to the first bout of immobility in the tail suspension test does not differ between hAPP and NTG mice. B, The total time spent immobile during the 6-minute tail suspension test trial is not significantly different between NTG and hAPP mice. C, There is no significant difference between hAPP and NTG mice in the time spent immobile during any individual minute of the trial. D, Repeated tail suspension trials reveal that NTG mice exhibit a significant decrease in latency to the first bout of immobility in the 24 hr trial relative to the baseline trial, but there is no change in the latency of hAPP mice in repeated trials. E, NTG mice spend more time immobile during a 24 hr tail suspension time trial relative to their baseline trial whereas hAPP mice do not change the time they spend immobile. F, Analyzing just the first three minutes of the tail suspension test reveals that hAPP mice spend less time immobile during the initial portion of the test. G, Analysis of the first three minutes of the tail suspension test at baseline and at the 24 hr trial confirms that NTG, but not hAPP, mice increase the time spent immobile upon repeated testing. n = 12/genotype (A–C) and n = 24/genotype (D–E). **p<0.01, ***p<0.001.

To determine whether impairments in adult neurogenesis correspond with the appearance of depressive behavior in 5–7 month old hAPP mice, we assessed depressive behavior using the tail suspension test. hAPP and NTG controls (n = 24/genotype) were tested in a trial lasting six minutes. For each mouse, we measured the latency to the first bout of immobility as well as the total time spent immobile during the six minute trial. Reduced latency to immobility and increased total time spent immobile during the tail suspension test are indicative of a depressive phenotype [22], [28]. At 5–7 months of age, hAPP and NTG mice exhibited similar latencies to the first bout of immobility (Fig. 2A). hAPP and NTG mice also exhibited similar total durations spent immobile during the six minute trial overall (Fig. 2B). Two-way repeated measures ANOVA on the time spent immobile on a minute by minute basis revealed an overall effect of genotype (F[1,23] = 4.855; p<0.05) and time (F[1,115] = 31.11; p<0.001), but none of the individual points were significant by Bonferroni post-hoc tests (Fig. 2C). There was no interaction effect (F[1,115] = 0.798; p = 0.55). Because 5–7 month old hAPP mice did not display depressive behavior in baseline tail suspension test trials, we examined whether a depressive phenotype might be revealed with repeated tail suspension test trials. Repeated testing in the tail suspension test induces a form of learned helplessness that can be measured as an increased magnitude of depressive behavior in subsequent trials [22], [28]. Consistent with this observation, NTG mice exhibited decreased latency to the first bout of immobility when tested 24 hours after a baseline trial; however, hAPP mice did not (Fig. 2D, n = 24/genotype). Two-way repeated measures ANOVA revealed an interaction effect between time and genotype, demonstrating that NTG mice reduced their latency on the 24 hr trial relative to baseline, but hAPP mice did not (F[1,46] = 13.46, p<0.001). One-way ANOVA followed by Newman-Keuls multiple comparisons tests demonstrated that there was an effect overall (F[3,92] = 13.40; p<0.001) and the decrease in latency exhibited by NTG mice was significant (p<0.001) but the slight decrease in hAPP mice was not (p>0.05). Similarly, NTG mice exhibited increased total immobility time over the six minute trial when tested 24 hours after a baseline trial, whereas hAPP mice did not (Fig. 2E). Again, two-way repeated measures ANOVA revealed an interaction between time and genotype (F[1,46] = 11.90, p<0.005). One-way ANOVA followed by Newman-Keuls multiple comparisons tests demonstrated that there was an effect overall (F[3,92] = 7.214; p<0.001), and the decrease in latency exhibited by NTG mice was significant (q = 4.724, p<0.01) but there was no change in hAPP mice (p>0.05). These results demonstrate that 5–7 month old hAPP mice do not exhibit depressive behavior observable with the tail suspension test either at baseline or with repeated testing.

We also examined the total immobility during only the first three minutes of the tail suspension test, because the minute-by-minute analysis in Fig. 2C indicated a separation between the curves of NTG and hAPP mice during this time. When we examined the total immobility over the first three minutes of the test, we found that hAPP mice actually spent less time immobile over these first three minutes relative to NTG mice (Fig. 2F; t[23] = 2.185, p<0.05).

We then examined whether this difference would affect the measurement of learned helplessness in repeated testing. When we re-analyzed the data using only the first three minutes of the tail suspension test at both baseline and 24 hr trials, we confirmed that NTG mice exhibited increased total immobility time when tested 24 hours after a baseline trial, whereas hAPP mice did not (Fig. 2G). Two-way repeated measures ANOVA revealed effects of both time and genotype (time: F[1,23] = 20.5, p<0.001; genotype: F[1,23] = 15.76, p<0.001). There was no interaction effect (F[1,23] = 2.909, p = 0.1). This result suggests that both NTG and hAPP mice similarly altered their behavior at the 24 hr test relative to baseline. However, one-way ANOVA followed by Newman-Keuls multiple comparisons tests demonstrated that there was an effect overall (F[3,46] = 12.00; p<0.001), and that the increase in immobility exhibited by NTG mice at the 24 hour test was significant (q = 4.874, p<0.01) but there was no significant increase in immobility in hAPP mice at the 24 hr test (p>0.05).

### Hippocampal-dependent memory is impaired in 5–7 month old hAPP mice

**Figure 3:**
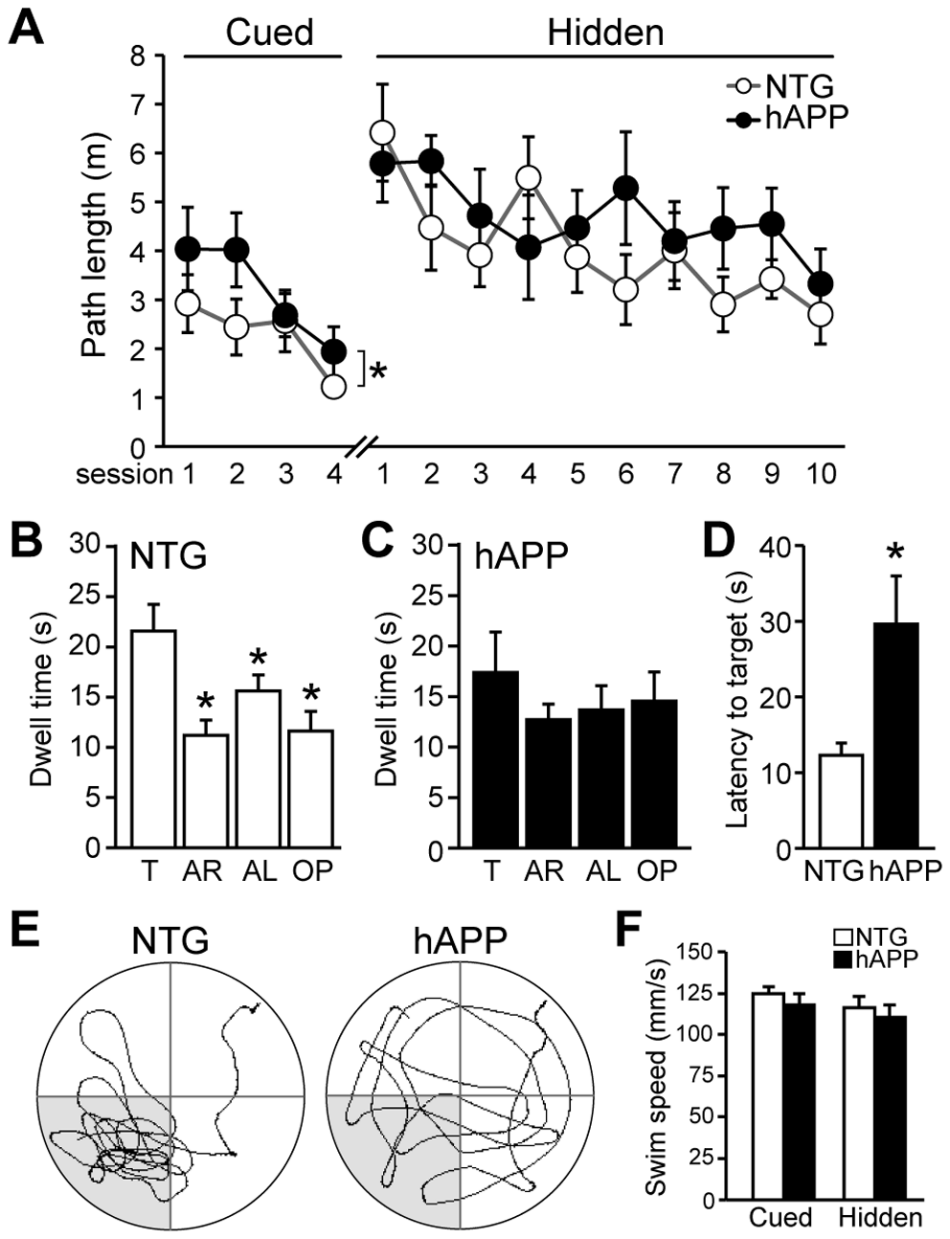
Hippocampal-dependent memory deficits are observed in 5–7 month old hAPP mice. A, Performance in the acquisition portion of the Morris water maze. In the cued platform portion, 2-way repeated measures ANOVA indicates an overall difference between NTG and hAPP mice (p<0.05), but no individual point reached significance in post-hoc tests. There was no significant difference between NTG and hAPP mice in the hidden platform portion of the water maze. B–C, During the probe trial test for memory that occurred 24 hrs after the last training trial of the hidden platform acquisition phase, NTG mice spent more time in the target quandrant relative to the other three quadrants (B) whereas hAPP mice did not (C). D, The latency for hAPP mice to reach the target area for the first time during the probe trial was higher than that for NTG mice. E, Representative paths swum during the probe trial for a NTG and an hAPP mouse. F, Swim speed was similar for NTG and hAPP mice during both the cued and hidden platform portions of the water maze. Swim speeds were compared by assessing the average swim speeds over all sessions in the cued or hidden platform portions of the test. T, target; AR, adjacent right; AL, adjacent left; OP, opposite. n = 12/genotype. *p<0.05.

Adult neurogenesis in the hippocampus is important for both cognition as well as mood [9]–[11]. We and others have previously demonstrated that several different lines of hAPP mice exhibit hippocampal-dependent memory deficits [23]–[26]. To confirm that 5–7 month old hAPP mice do exhibit memory impairments, we tested mice in the Morris water maze. Mice were trained first in the cued platform portion of the water maze, and then in the hidden platform portion of the water maze, when the platform is submerged beneath the surface of the water. In the acquisition phase of the water maze (Fig. 3A), we found that hAPP mice were impaired in the cued platform portion the Morris water maze (F[1,22] = 6.676, p<0.05) although no individual points reached statistical difference in Bonferroni post hoc tests; but hAPP and NTG mice did not differ in the hidden platform acquisition phase (F[1,22] = 3.973, p = 0.22). However, whereas NTG mice exhibited a preference for the target quadrant relative to the other three quadrants when tested in the probe trial, hAPP mice did not (Fig. 3B–C, E; F[3,44] = 7.799, p<0.001 for NTG; F[3,44] = 0.8519 for hAPP). In addition, the latency for hAPP mice to reach the target area for the first time was higher than that for NTG mice (Fig. 3D; t[22] = 2,652, p<0.05). There were no differences in swim speed between NTG and hAPP mice, either in the cued or hidden platform portions of the test, suggesting that hAPP mice have no overt sensorimotor deficits (Fig. 3F; CUED t[22] = 0.83, p = 0.415; HIDDEN t[22] = 0.61, p = 0.55). Swim speeds were compared by assessing the average swim speeds over all sessions in the cued or hidden platform portions of the test. These results confirm that hAPP mice at this age have memory impairments.

### Impairments in hippocampal neurogenesis in hAPP mice at 13–15 months of age

**Figure 4:**
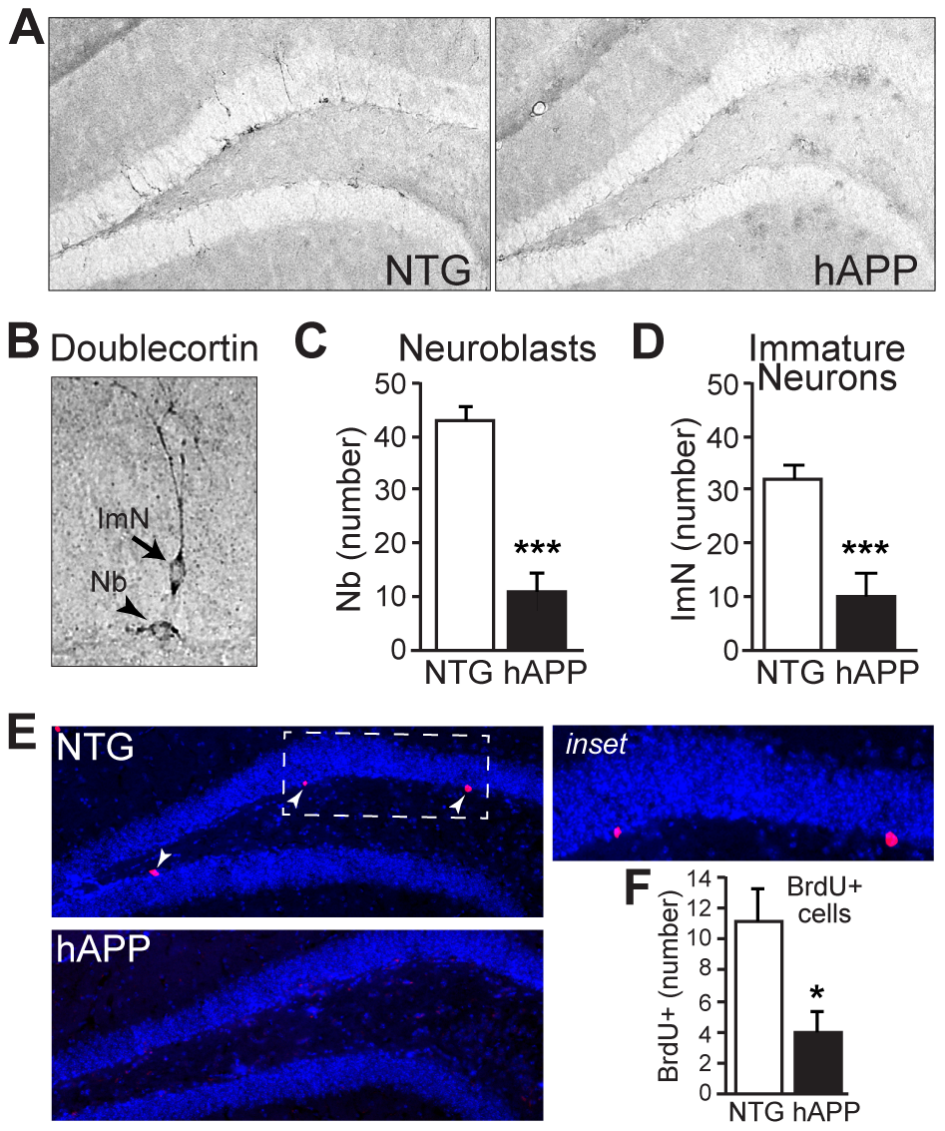
13–15 month old hAPP mice exhibit decreased neurogenesis. A, Micrographs of doublecortin immunostaining in coronal brain sections from NTG and hAPP mice. B, High magnification micrograph illustrating doublecortin-positive neuroblasts (Nb) and immature neurons (ImN). C–D, Quantification of doublecortin expression demonstrates significant decreases in neuroblasts (C) and immature neurons (D) in hAPP mice relative to NTG controls. E–F, BrdU labeling of dividing cells in the subgranular zone demonstrates fewer dividing cells in the subgranular zone of hAPP mice. Arrowheads, BrdU-labeled cells. n = 11–12/genotype. *p<0.05, ***p<0.001.

We then examined doublecortin expression in coronal sections from 13–15 month old hAPP mice to assess neurogenesis later in disease progression, when both behavioral/cognitive deficits and extracellular plaques are evident in hAPP mice [25], [26]. At this age NTG mice continue to demonstrate ongoing adult neurogenesis in the hippocampus, although the number of neuroblasts and immature neurons decreases with age, as expected (compare Fig. 4A–D to Fig. 1A–D, see Table 1). For neuroblasts, there was an average of 268.8±12.02 in 5–7 month old mice vs 43±2.6 neuroblasts in 13–15 month old NTG mice (t[22] = 18.35, p<0.001). For immature neurons, there was an average of 232.2±11.7 in 5–7 month old mice vs a 32.08±2.4 immature neurons in 13–15 month old NTG mice (t[22] = 16.67, p<0.001). Neuroblasts and immature neurons maintain the same cell morphology regardless of age (compare Fig. 4B to Fig. 1B). Notably, relative to age-matched NTG controls, 13–15 month old hAPP mice exhibit significant decreases in both neuroblasts (t[21] = 7.527, p<0.001) and immature neurons (t[21] = 4.489, p<0.001) in the dentate gyrus (Fig. 4C–D; n = 11–12/genotype).

**Table 1:** Age-related changes in doublecortin-expressing cells.

	5–7 mo old	13–15 mo old	p-value
**Neuroblasts:**			
NTG vs NTG (number of cells)	268.8±12.02	43±2.6	t[22] = 18.35, p<0.001
hAPP vs hAPP (number of cells)	134±16.06	10.91±3.42	t[22] = 7.209, p<0.001
hAPP vs hAPP (rel. to age-matched NTG)	0.5±0.06	0.25±0.08	t[21] = 2.50, p<0.05
**Immature Neurons:**			
NTG vs NTG (number of cells)	232.2±11.7	32.08±2.4	t[22] = 16.67, p<0.001
hAPP vs hAPP (number of cells)	112.5±19.77	10±4.44	t[21] = 4.854, p<0.001
hAPP vs hAPP (rel. to age-matched NTG)	0.485±0.085	0.312±0.138	t[21] = 1.085, p = 0.29

Numbers of neuroblasts and immature neurons in each age group and genotype are listed for comparative purposes. Three comparisons are provided: 1) numbers of cells in 5–7 mo old NTG mice vs 13–15 mo old NTG mice; 2) numbers of cells in 5–7 mo old hAPP mice vs 13–15 mo old hAPP mice; and 3) the proportion of cells in 5–7 mo old hAPP mice relative to age-matched NTG mice vs the propotion of cells in 13–15 mo old hAPP mice relative to age-matched NTG mice.

We also found that the degree of neurogenesis impairments in hAPP mice (relative to age-matched NTG controls) appeared to increase with age. We calculated the level of neuroblasts and immature neurons in hAPP mice as a percentage of age-matched NTG controls in each age group, and then compared the relative values of hAPP mice in the two age groups. The reduction in neuroblasts is greater in 13–15 month old hAPP mice compared to 5–7 month old hAPP mice (Table 1; t[21] = 2.5, p<0.05). The reduction in immature neurons also appears to be greater in 13–15 month hAPP mice, although this did not reach statistical significance (Table 1; t[21] = 1.085, p = 0.29).

To confirm that the reductions in doublecortin-expressing cells in 13–15 month old hAPP mice are due to reduced cell proliferation as exhibited by the 5–7 month old hAPP mice, we examined the incorporation of BrdU into cells in the subgranular zone of the dentate gyrus of 13–15 month old hAPP and NTG mice. We found that the numbers of BrdU-labeled cells was reduced in hAPP mice relative to NTG controls (Fig. 4E–F; t[21] = 2.738, p<0.05).

### hAPP mice exhibit a subtle depressive phenotype at 13–15 months of age

**Figure 5:**
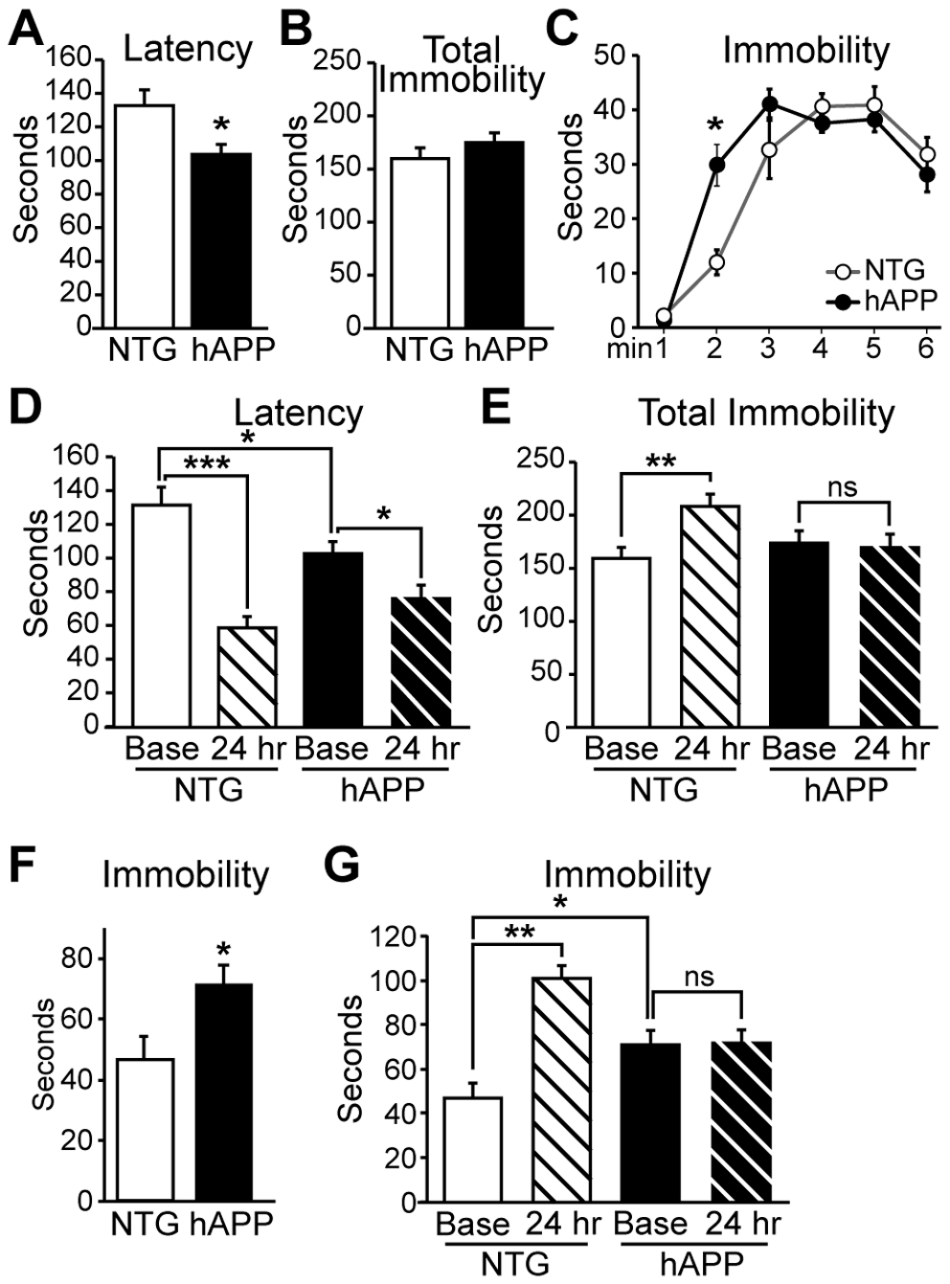
13–15 month old hAPP mice display subtle depressive behavior. A, In the tail suspension test, hAPP mice exhibit decreased latency to the first bout of immobility relative to NTG controls. B, No significant difference between hAPP and NTG mice in the total time spent immobile during the 6-minute tail suspension test trial. C, hAPP mice become immobile sooner than do NTG mice during the tail suspension trial. D, Both NTG and hAPP mice demonstrate decreased latency to the first bout of immobility in response to a repeated tail suspension test. E, NTG mice increase the time spent immobile during a repeated tail suspension test, whereas there is no change in hAPP mice. F, Analyzing just the first three minutes of the tail suspension test reveals that hAPP mice spend more time immobile during the initial portion of the test. G, Analysis of the first three minutes of the tail suspension test at baseline and at the 24 hr trial confirms that NTG, but not hAPP, mice increase the time spent immobile upon repeated testing. n = 11–12/genotype. *p<0.05, ** p<0.01, ***p<0.001.

We tested 13–15 month old hAPP and NTG mice with the tail suspension test to assess whether the exacerbated deficits in neurogenesis evident in 13–15 month old hAPP mice with advanced disease progression are associated with depressive behavior. 13–15 month old hAPP mice demonstrated shorter latencies to their first bouts of immobility relative to NTG controls (Fig. 5A; t[21] = 2.559, p<0.05). Although the total time spent immobile during the 6 minute trial did not differ between NTG and hAPP mice (Fig. 5B; t[21] = 1.196, p = 2.449), analysis of the time spent immobile on a minute-by-minute basis revealed that hAPP mice became immobile sooner than did NTG mice (Fig. 5C). Two-way repeated measures ANOVA revealed an interaction between time and genotype (F[1,21] = 5.057, p<0.001), and Bonferroni post hoc test demonstrated that hAPP mice spend more time immobile already at the second minute of the six minute trial (p<0.001).

To determine whether the depressive phenotype in hAPP mice could be exacerbated by repeated testing in the tail suspension test, we assessed hAPP and NTG mice in a second tail suspension test 24 hours after the baseline trial. At 13–15 months of age, both NTG and hAPP mice mice demonstrated decreased latency to the first bout of immobility on the 24 hour tail suspension test relative to their baseline trials (Fig. 5D). One-way ANOVA followed by Neuman-Keuls multiple comparisons tests demonstrated that the decrease in latency in both genotypes was significant (F[3,42] = 19.32, p<0.001 overall; p<0.001 for NTG; p<0.05 for hAPP). However, two-way repeated measures ANOVA revealed that time, but not genotype, induced a significant effect on performance (F[1,21] = 50.91, p<0.001), which may reflect the larger effect in NTG mice relative to hAPP mice. Indeed, there was an interaction effect, demonstrating that time affected the two genotypes differently (F[1,21] = 10.75, p<0.01). When we assessed the total time spent immobile on the 24 hour trial relative to the baseline trial, only NTG mice increased the time spent immobile (Fig. 5E). Two-way repeated measures ANOVA showed that there was a significant interaction effect between time and genotype (F[1,21] = 21.75, p<0.001). One-way ANOVA followed by Neuman-Keuls multiple comparisons tests demonstrated that the increase in immobility was significant for NTG but not for hAPP mice (F[3,42] = 4.819, p<0.01 overall; p<0.01 for NTG; p>0.05 for hAPP). Together these results indicate that 13–15 month old hAPP mice exhibit some, but not all, measures of increased depressive behavior with repeated testing.

Because the minute-by-minute analysis in Fig. 5C indicated a separation between the curves of NTG and hAPP mice during the first three minutes of the tail suspension test, we also examined the total immobility during only the first three minutes. We found that hAPP mice spent more time immobile over these first three minutes relative to NTG mice (Fig. 2F; t[21] = 2.66, p<0.05), indicating that hAPP mice exhibit a subtle depressive phenotype at 13–15 months of age, an age at which neurogenesis is greatly impaired.

Since hAPP mice did exhibit depressive behavior when analyzing the initial portion of the tail suspension test, we then examined whether analysing this portion of the test would reveal a learned helplessness phenotype in hAPP mice with repeated testing. When we re-analyzed the data using only the first three minutes of the tail suspension test at both baseline and 24 hr trials, we confirmed that NTG mice exhibited increased total immobility time when tested 24 hours after a baseline trial. However, hAPP mice did not (Fig. 2G). Two-way repeated measures ANOVA showed that there was a significant interaction effect between time and genotype (F[1,21] = 43.72, p<0.001). One-way ANOVA followed by Neuman-Keuls multiple comparisons tests demonstrated that the increase in immobility was significant for NTG but not for hAPP mice (F[3,42] = 13.57, p<0.001 overall; p<0.001 for NTG; p>0.05 for hAPP). Together these results indicate that although 13–15 month old hAPP mice exhibit depressive behaviors, they do not exhibit a learned helpessness phenotype with repeated testing.

### Hippocampal-dependent memory is impaired in 13–15 month old hAPP mice

**Figure 6:**
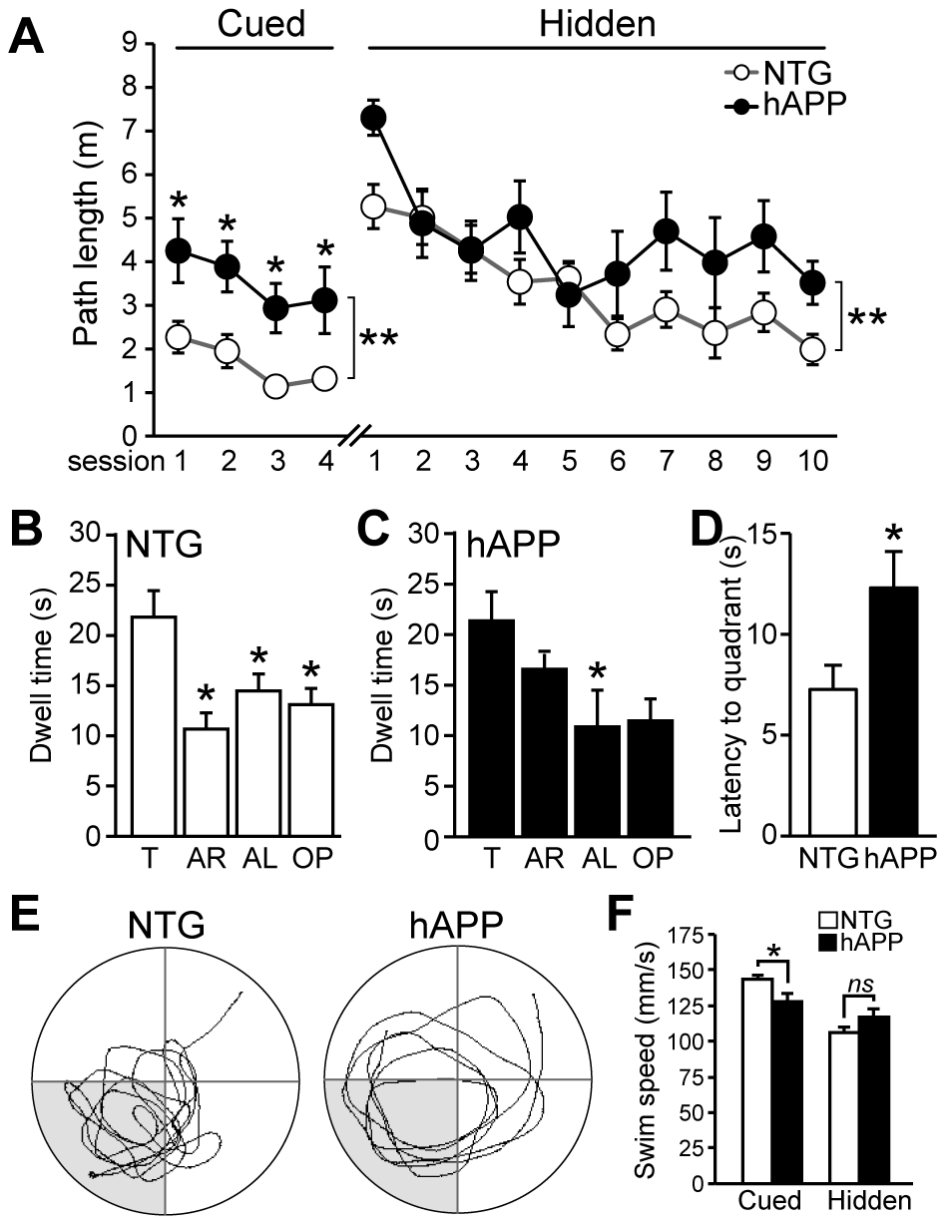
Hippocampal-dependent memory deficits are observed in 13–15 month old hAPP mice. A, Performance in the acquisition portion of the Morris water maze. In the cued platform portion, 2-way repeated measures ANOVA indicates an overall difference between NTG and hAPP mice (p<0.01). Asterisk denotes p<0.05 for individual data points. There was also a significant difference between NTG and hAPP mice in the hidden platform portion of the water maze, although no individual point reached significance in post-hoc tests (2-way repeated measures ANOVA p<0.01). B–C, During the probe trial test for memory that occurred 24 hrs after the last training trial of the hidden platform acquisition phase, NTG mice spent more time in the target quandrant relative to the other three quadrants (B) whereas hAPP mice did not (C). D, The latency for hAPP mice to reach the correct quadrant for the first time during the probe trial was higher than that for NTG mice. E, Representative paths swum during the probe trial for a NTG and an hAPP mouse. F, Swim speed was lower for hAPP mice than NTG mice in the cued portion of the water maze, but was similar to NTG mice during the hidden platform portion of the maze. Swim speeds were compared by assessing the average swim speeds over all sessions in the cued or hidden platform portions of the test. n = 7–11/genotype. T, target; AR, adjacent right; AL, adjacent left; OP, opposite. *p<0.05, **p<0.01.

We also tested 13–15 month old NTG and hAPP mice in the Morris water maze to assess hippocampal-dependent spatial memory. We found that hAPP mice were impaired in the cued portion of the water maze (Fig. 6A), although they did exhibit learning throughout the acquisition sessions (F[1,16] = 24.61, p<0.001). hAPP mice were also significantly impaired in acquisition of the hidden platform portion of the water maze (F[1,16] = 9.653, p<0.01). Notably, NTG mice performed well in the probe trial (Fig. 6B, E), spending more time in the target quadrant relative to the other three quadrants (F[3,40] = 6.291, p<0.005; Tukey post-hoc tests p<0.05). However, hAPP mice exhibited memory impairment in the probe trial (Fig. 6C, E). Although two-way repeated measures ANOVA indicated an overall difference (F[3,24] = 3.532, p<0.05), Tukey post-hoc tests revealed that hAPP mice only exhibited a preference for the target quadrant relative to the adjacent left quadrant, but not to the other two quadrants (p<0.05 vs adjacent left; p>0.05 vs adjacent right and opposite quadrants). We also found that the latency for hAPP mice to reach the correct quadrant area for the first time was higher than that for NTG mice (Fig. 6D; t[16] = 2.43, p<0.05). These results indicate that hippocampal-dependent memory impairments persist with age in hAPP mice. We found that although the swim speed of hAPP mice was slower than that of NTG mice in the cued portion of the water maze (Fig. 6F; t[1,16] = 2.62, p<0.05), there was no difference in swim speed between NTG and hAPP mice in the hidden platform portion of the test (Fig. 6E; t[1,16] = 1.45, p = 0.167). Because swim speed was similar in the hidden platform portion of the test, this indicates that there is likely no overt sensorimotor deficits in hAPP mice, but suggests that other factors may have affected performance in the cued portion of the test.

## Discussion

In this report, we demonstrate that hAPP mice exhibit significant impairments in neurogenesis by 5–7 months of age that worsen with age, but subtle depressive behavior in the tail suspension test is only measurable by 13–15 months of age. However, hAPP mice exhibit impairments in spatial memory already by 5–7 months of age. We and others have observed memory deficits in several versions of the water maze in this line of mice [23], [29]–[32], but a comparison to the development of depressive behaviors has not previously been described.

The fact that no depressive behavior is evident at 5–7 months, when hAPP mice already display significant impairments in neurogenesis, suggests that these phenotypes may not be tightly linked in hAPP mice. Indeed, hAPP mice exhibited less immobility in the first half of the tail suspension test, consistent with a less depressive phenotype. Even with repeated testing in the tail suspension test, hAPP mice did not show a decrease in latency to immobility nor an increase in total immobility as did NTG mice, which is again consistent with a less depressive phenotype. Older hAPP mice at 13–15 months of age did exhibit depressive behaviors. However, because the depressive behavior exhibited by 13–15 month old hAPP mice is associated with greater deficits in neurogenesis than are evident at 5–7 months, it is possible that more advanced impairments in neurogenesis are necessary before the development of a depressive phenotype. Notably, although 13–15 month old hAPP mice exhibited a depressive phenotype, they did not exhibit learned helpessness with repeated testing in the tail suspension test, which may be a indication of a failure to adapt behavior as a result of experience. Indeed, it is noteworthy that whether hAPP mice spend less time immobile during the first 3 minutes of the trial (as for the 5–7 month old mice, Fig. 2F) or whether they spend more time immobile during the first 3 minutes of the trial (as for the 13–15 month old mice, Fig. 5F), hAPP mice do not exhibit any differences between the first and second tail suspension tests, whereas NTG mice do (Fig. 2G and 5G).

If advanced deficits in neurogenesis in hAPP mice are required for the development of a depressive phenotype, then how might these impairments be related to depression in AD, which often manifests early in disease progression [33], [34]? Although deficits in neurogenesis are associated with depression, reduced neurogenesis alone is not sufficient to induce depression in rodent models [16], [17]. However, early deficits in neurogenesis in AD may allow other pathological symptoms of the disease to cause depression. If reduced neurogenesis acts as a permissive factor for the development of depression, and impairments in neurogenesis worsen with age and disease progression, then more advanced deficits in neurogenesis may be present by the time depression manifests in AD. Consistent with this possibility, the therapeutic effects of several classes of antidepressants rely on mechanisms that increase neurogenesis [16]–[18].

It is important to also consider the possibility that the tail suspension test may not be sensitive enough to detect a subtle depressive phenotype that might exist in early stages of disease in hAPP mice. The tail suspension test is widely used as a robust assay for assessing the therapeutic effects of antidepressants [28]; however, a more ethologically-relevant behavioral task may be better able to detect subtle mood disorders in our mice. To address this possibility, we also tested hAPP and NTG mice with the forced swim test [22], [28], as this test has been previously used to assess depressive behavior in an AD mouse model [35]. In our study, however, we found that results were complicated by anxiety-related behaviors exhibited by hAPP mice when placed in the task chamber (unpublished observations), making it difficult to interpret the data. hAPP mice did not exhibit any anxiety-related behaviors in the tail suspension test. A different type of ethologically-relevant behavioral paradigm such as the resident intruder test [36] may better assess mild depressive behavior in hAPP mice and reveal a depressive phenotype at a younger age. Regardless, our results indicate that although robust impairments in neurogenesis are observed early in disease progression in hAPP mice, a depressive phenotype is not overt, and reaches only subtle indications at older ages. Together these results suggest that depressive behaviors and impairments in neurogenesis and are not tightly linked in hAPP mice.

Whereas subtle depressive behavior is only evident in hAPP mice by 13–15 months of age, the inability of these mice to learn and modify their behavior in response to repeated tail suspension trials is apparent by 5–7 months of age. At this age (5–7 mo), hAPP mice also already exhibit spatial memory deficits in the water maze. Together, these observations underscore the fact that whereas depression is a common symptom of AD, the most prominent symptom of AD is cognitive deficit, particularly related to learning and memory [1], [33], [37].
